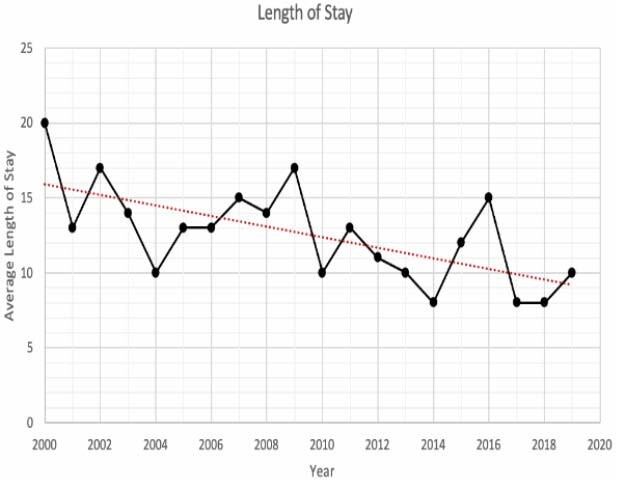# 537 Epidemiology of Elderly Burn Injuries – A Five Decade Single Center Retrospective Review

**DOI:** 10.1093/jbcr/irad045.134

**Published:** 2023-05-15

**Authors:** Elisa Emanuelli, Angela Li, Richard Korentager, Dhaval Bhavsar

**Affiliations:** The University of Kansas Medical Center, Kansas City, Kansas; The University of Kansas Medical Center, Kansas City, Kansas; The University of Kansas Medical Center, Kansas City, Kansas; University of Kansas Medical Center, Kansas City, Kansas; The University of Kansas Medical Center, Kansas City, Kansas; The University of Kansas Medical Center, Kansas City, Kansas; The University of Kansas Medical Center, Kansas City, Kansas; University of Kansas Medical Center, Kansas City, Kansas; The University of Kansas Medical Center, Kansas City, Kansas; The University of Kansas Medical Center, Kansas City, Kansas; The University of Kansas Medical Center, Kansas City, Kansas; University of Kansas Medical Center, Kansas City, Kansas; The University of Kansas Medical Center, Kansas City, Kansas; The University of Kansas Medical Center, Kansas City, Kansas; The University of Kansas Medical Center, Kansas City, Kansas; University of Kansas Medical Center, Kansas City, Kansas

## Abstract

**Introduction:**

Age has long been known to be an independent risk factor for worse outcomes in burn patients. In comparison to younger patients, geriatric patients have higher morbidity and mortality. As the median age of the US population has increased, we have seen a greater proportion of elderly burn patients at our burn unit over the last five decades.

**Methods:**

Retrospective chart review was performed for all burn patients admitted to our burn unit between 2000 and 2019. Demographics and outcomes data was collected for geriatric patients (age 65 and older). This was compared to historic data available for our burn unit between 1972 to 2000.

**Results:**

Between 2000 to 2019, our burn unit admitted a total of 5881 burn patients of all ages. There were 652 (11%) geriatric patients admitted. The proportion of geriatric patients admitted statistically significantly increased over that period. Meanwhile, length of stay and mortality statistically significantly decreased in the 2000-2019 time period compared to the previous period. The main mechanism of burn injury was flame exposure. Compared to data on geriatric burn patients from 1972 to 2000, mortality has decreased substantially. In the 1970s mortality was 77% compared to 21% in the past decade. The mortality rate increased significantly with age, TBSA and inhalation injury over both time periods.

**Conclusions:**

Though lagging behind younger age groups, overall survival and outcomes are improving in elderly burn patient due to advances in critical care and surgical burn management. Acknowledging the unique characteristics of this group of patients, such as impaired healing and multiple comorbidities, allows for improved outcomes.

**Applicability of Research to Practice:**

Burn centers should notice and prepare for an increasing trend in elderly burn patient admission.